# Medication Use in the Management of Comorbidities Among Individuals With Autism Spectrum Disorder From a Large Nationwide Insurance Database

**DOI:** 10.1001/jamapediatrics.2021.1329

**Published:** 2021-06-07

**Authors:** Aliya G. Feroe, Nishant Uppal, Alba Gutiérrez-Sacristán, Sajad Mousavi, Philip Greenspun, Rajeev Surati, Isaac S. Kohane, Paul Avillach

**Affiliations:** 1 Harvard Medical School, Boston, Massachusetts; 2 Department of Biomedical Informatics, Harvard Medical School, Boston, Massachusetts; 3 Computational Health Informatics Program, Boston Children’s Hospital, Boston, Massachusetts

## Abstract

**Importance:**

Although there is no pharmacological treatment for autism spectrum disorder (ASD) itself, behavioral and pharmacological therapies have been used to address its symptoms and common comorbidities. A better understanding of the medications used to manage comorbid conditions in this growing population is critical; however, most previous efforts have been limited in size, duration, and lack of broad representation.

**Objective:**

To use a nationally representative database to uncover trends in the prevalence of co-occurring conditions and medication use in the management of symptoms and comorbidities over time among US individuals with ASD.

**Design, Setting, and Participants:**

This retrospective, population-based cohort study mined a nationwide, managed health plan claims database containing more than 86 million unique members. Data from January 1, 2014, to December 31, 2019, were used to analyze prescription frequency and diagnoses of comorbidities. A total of 26 722 individuals with ASD who had been prescribed at least 1 of 24 medications most commonly prescribed to treat ASD symptoms or comorbidities during the 6-year study period were included in the analysis.

**Exposures:**

Diagnosis codes for ASD based on *International Classification of Diseases, Ninth Revision*, and *International Statistical Classification of Diseases and Related Health Problems, Tenth Revision.*

**Main Outcomes and Measures:**

Quantitative estimates of prescription frequency for the 24 most commonly prescribed medications among the study cohort and the most common comorbidities associated with each medication in this population.

**Results:**

Among the 26 722 individuals with ASD included in the analysis (77.7% male; mean [SD] age, 14.45 [9.40] years), polypharmacy was common, ranging from 28.6% to 31.5%. Individuals’ prescription regimens changed frequently within medication classes, rather than between classes. The prescription frequency of a specific medication varied considerably, depending on the coexisting diagnosis of a given comorbidity. Of the 24 medications assessed, 15 were associated with at least a 15% prevalence of a mood disorder, and 11 were associated with at least a 15% prevalence of attention-deficit/hyperactivity disorder. For patients taking antipsychotics, the 2 most common comorbidities were combined type attention-deficit/hyperactivity disorder (11.6%-17.8%) and anxiety disorder (13.1%-30.1%).

**Conclusions and Relevance:**

This study demonstrated considerable variability and transiency in the use of prescription medications by US clinicians to manage symptoms and comorbidities associated with ASD. These findings support the importance of early and ongoing surveillance of patients with ASD and co-occurring conditions and offer clinicians insight on the targeted therapies most commonly used to manage co-occurring conditions. Future research and policy efforts are critical to assess the extent to which pharmacological management of comorbidities affects quality of life and functioning in patients with ASD while continuing to optimize clinical guidelines, to ensure effective care for this growing population.

## Key Points

**Question:**

How have medications been used recently in the management of symptoms and comorbidities of autism spectrum disorder (ASD)?

**Findings:**

This population-based cohort study of 26 722 individuals with ASD in the US retrospectively assessed the most common medications used to treat their symptoms and comorbidities during 6 years. Medication type and frequency varied considerably, depending on the comorbidity diagnosed, and individual drug regimens shifted frequently within medication classes over time.

**Meaning:**

Many individuals with ASD undergo treatment with a wide variety of medications on a trial basis, resulting in frequent changes in drug regimens over time as clinicians attempt to manage associated symptoms and comorbidities.

## Teaser

This population-based cohort study assesses trends in the prevalence of co-occurring conditions and medication use in the management of symptoms and comorbidities over time among US individuals with autism spectrum disorder.

## Introduction

In 2016, approximately 1.9% (1 in 54) of US children 8 years of age were diagnosed with autism spectrum disorder (ASD),^1^ and the prevalence has increased since then. That growth has spurred more expansive efforts to understand not only the prevalence of the disease but also its comorbidities and corresponding pharmacological treatments.^1,2^

Management of ASD has focused primarily on behavioral and educational interventions, which address core deficiencies in social communication and repetitive patterns of behavior. Although pharmacological intervention is not intended to reverse ASD-related disabilities, medications can treat symptoms of ASD and co-occurring conditions, including intellectual disabilities, language delays, attention-deficit/hyperactivity disorder (ADHD), anxiety, depression, agitation, irritability, disruptive behavior, and sleep disorders.^3,4,5,6,7,8,9^

At least 83% of children and adolescents with ASD in the US have at least 1 co-occurring developmental disorder, and 70% display a co-occurring psychiatric condition.^8,10^ Physicians have treated these comorbidities with pharmaceutical agents, with varying degrees of success.^3,6,7,9^

Overall, pharmacological agents used to treat patients with ASD fall into 3 broad categories, each of which is based on the symptoms targeted: (1) agitation and irritation; (2) hyperactivity, impulsivity, and inattentive-type ADHD; and (3) mood and anxiety disorders, including major depressive disorder and obsessive-compulsive disorder.^11,12^ The major psychotropic medication classes used in individuals with ASD include ADHD medications (stimulants, α_2_-adrenergic agonists, and neurotransmitter modulators),^13,14^ antipsychotics,^15,16^ antidepressants,^17,18,19,20^ mood stabilizers, benzodiazepines, anxiolytics, and hypnotics.^2^

The prescription rates of these medications for patients with ASD have not been clearly established.^21^ Some studies^22,23,24,25,26^ have estimated that 30% to 50% of patients with ASD have been treated with at least 1 medication. However, these estimates may be unreliable because they were derived from studies that depended primarily on patient surveys; have not been replicated after shifts in diagnostic criteria for ASD; and/or predate the establishment of the Affordable Care Act and other significant changes to prescription drug coverage.^22,23,24,25,26^ Despite these limitations, however, the use of pharmacotherapy in patients with ASD appears to be growing rapidly; the prescription rate of stimulants to treat co-occurring ADHD and ASD increased 5-fold from 1990 to 2001.^27^

Clinicians caring for patients with ASD are tasked with the challenges of managing the primary disease, as well as co-occurring medical conditions, and coordinating with educational and social service professionals to provide holistic care. Furthermore, engaging in shared decision-making with patients and families to develop the medical home and support it longitudinally creates additional pressures for primary care clinicians. Recent work has described diagnostic overshadowing, whereby clinicians conflate patients’ ASD traits with signs of a comorbidity, and vice versa.^28^ Such behavior can influence complex pharmacotherapy regimens to manage these conditions.^28^ Although clinician awareness of co-occurring conditions has broadened in recent years, we hypothesize that the difficulties in adequately managing the symptoms of ASD contribute to high variability in pharmacotherapy use, even for patients whose co-occurring conditions have been diagnosed.

Previous studies of co-occurring conditions and use of psychotropic medication in individuals with ASD,^20,22,23,26^ though informative, have been limited to smaller, regional study populations. Our objective was to evaluate the consistency of drug prescriptions for symptoms and comorbidities related to ASD using US insurance claims data and to determine the true extent of the secondary burden of disease and identify patterns in pharmacological treatment that can inform clinical care.

## Methods

### Data Source

This population-based, retrospective cohort study was conducted using a US national managed care plan claims database. The database contains demographic and enrollment information from January 1, 2008, to December 31, 2019, for 86 million members of a large national health plan. The data set contains inpatient claims, outpatient claims, and records of individual prescription transactions filled by commercial pharmacies. The study was deemed exempt from institutional review board approval and informed consent by Harvard Medical School, Boston, Massachusetts, because all data were deidentified. This study followed the Reporting of Studies Conducted Using Observational Routinely Collected Health Data (RECORD) guidelines^29^ (eMethods 2 in the Supplement), an extension of the existing Strengthening the Reporting of Observational Studies in Epidemiology (STROBE) reporting guideline for cohort studies.^30^

### Study Population

**Figure 1.**
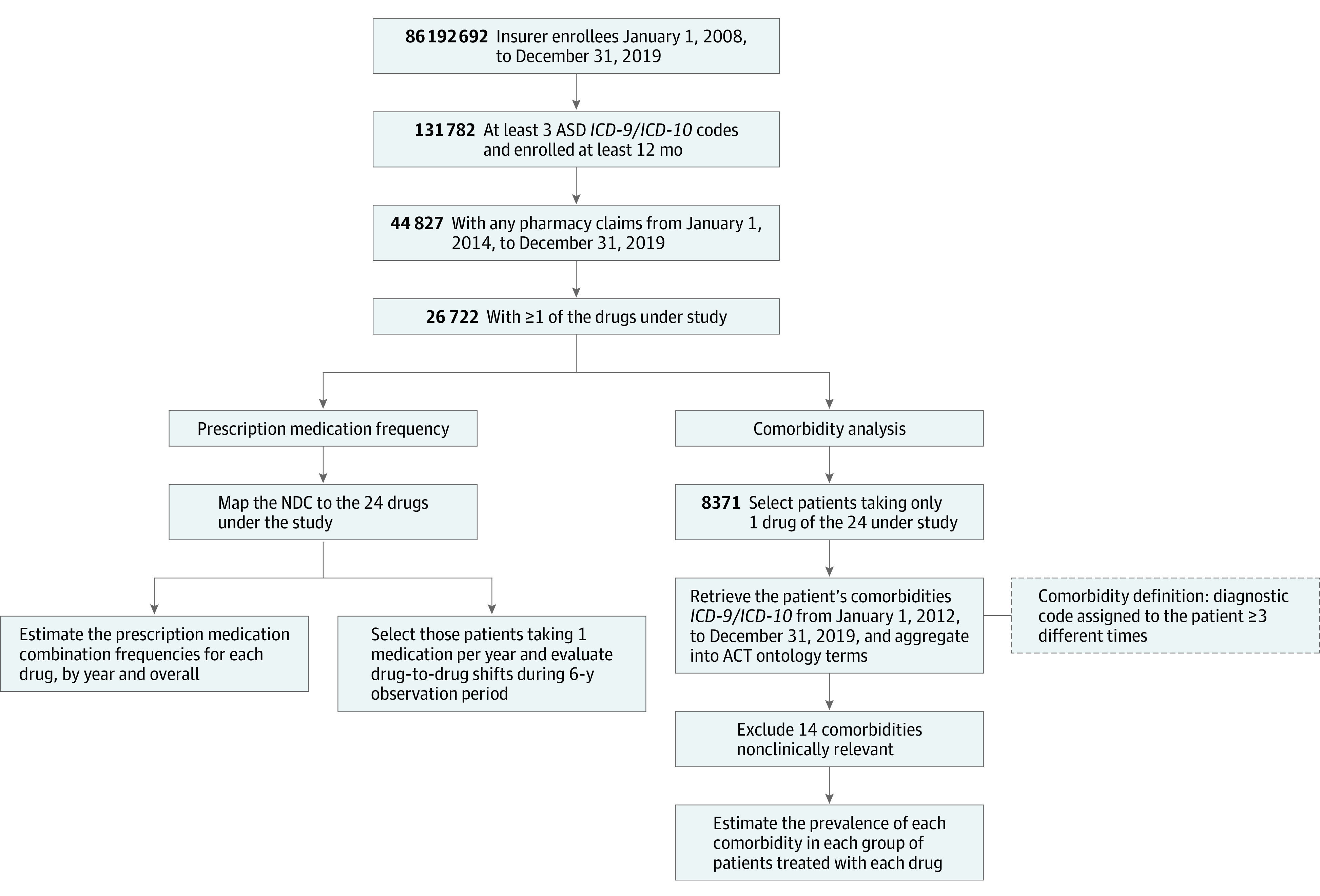
Workflow for Cohort Development and Data Analysis ACT indicates Accrual to Clinical Trials; ASD, autism spectrum disorder; ICD- 9, *International Classification of Diseases, Ninth Revision*; ICD-10, *International Statistical Classification of Diseases and Related Health Problems, Tenth Revision*; and NDC, National Drug Codes.

Following methods from existing studies, we identified all members in the claims database with at least 3 distinct diagnostic codes for ASD, based on *International Classification of Diseases, Ninth Revision* (*ICD-9*),^31^ and *International Statistical Classification of Diseases and Related Health Problems, Tenth Revision* (*ICD-10*)^32^ (*ICD-9* codes 299.0, 299.00, 299.01, 299.8, 299.81, 299.9, 299.90, 299.91, 299, 299.1, 299.10, and 299.11; *ICD-10* codes F84, F84.0, F84.3, F84.5, and F84.9)^2,33^ during the 6-year period from January 1, 2014, to December 31, 2019 (Figure 1).

### Prescription Medication Frequency

We retrieved deidentified pharmaceutical data for each member of the ASD cohort by mapping the various National Drug Code descriptions within the data set to the generic drug name for each of the 24 study drugs. We estimated the frequency of prescription medication combinations for each drug, annually and overall (eg, number of patients prescribed with methylphenidate hydrochloride, number of patients prescribed with methylphenidate and guanfacine hydrochloride).

To evaluate drug-to-drug shifts within individuals’ ASD-related medication regimens during the 6-year observation period, we extracted the prescription year for each drug under study, patient by patient. We restricted our analysis of changes in medication use to members who were enrolled nearly continuously during the 6 years. To simplify interpretation, we considered only members using 1 of the study drugs in any given year and estimated medication changes over time.

### Comorbidity Analysis

Using the Accrual to Clinical Trials (ACT) ontology, version 2.0.1, we retrieved data on patient comorbidities (diagnosed from January 1, 2012, to December 31, 2019) in individuals using 1 of the study drugs in any given year.^34,35^ Diagnostic data were intentionally retrieved 2 years before the beginning of the 6-year study period (2014-2019) to ensure that diagnoses of comorbidities preceded the prescription of the medications under study. We aggregated the 31 701 *ICD-9* and *ICD-10* codes into 7323 level 3 ACT terms (a detailed explanation of ACT is provided in eMethods 1 in the Supplement).

We only included comorbid conditions in the analysis if the individual had at least 3 distinct diagnoses of the given comorbidity. We used the ACT terms to estimate the prevalence of each comorbidity in individuals treated with 1 of the drugs under study. We removed comorbidities that were not considered clinically relevant for this study. The workflow is depicted in Figure 1, and the complete list of excluded comorbidities is provided in the eTable in the Supplement.

All data analyses were performed using Microsoft SQL Server, version 17.9 (Microsoft Corporation), and R, version 3.4.1 (R Program for Statistical Computing). The code to reproduce these analyses is available on GitHub (https://github.com/hms-dbmi/medicationUsageASDcomorbidities).

## Results

### Cohort Characteristics

Of the 86 192 692 total health plan enrollees available for this analysis, 146 433 (0.2%) had at least 3 distinct diagnostic codes for ASD. After restricting the study period (for the purpose of analyzing medication trends), 44 827 members had available pharmacy claims and were enrolled for a minimum of 12 months during this 6-year period. From this sample, 26 722 members (59.6%) had been prescribed at least 1 of the 24 medications most commonly used for the management of comorbid conditions in ASD (Figure 1). The final ASD cohort was predominantly male (77.7% vs 22.3% female; mean [SD] age, 14.45 [9.40] years), with nearly one-third aged 6 to 11 years (30.6%) and more than one-third aged 12 to 18 years (39.3%) (complete cohort characteristics are provided in the Table).

**Table.** Characteristics of 26 722 Study Cohort Individuals With ASD

Characteristic	No. (%) of participants
Sex	
Male	20 769 (77.7)
Female	5951 (22.3)
Unspecified	2 (0.01)
Age, y^a^	
0-2	38 (0.1)
3-5	1475 (5.5)
6-11	8189 (30.6)
12-18	10 490 (39.3)
≥18	6530 (24.4)
Geographic region^b^	
West	4137 (15.5)
Midwest	3972 (14.9)
Southwest	3663 (13.7)
Southeast	6320 (23.7)
Northeast	8144 (30.5)
Noncontiguous	225 (0.8)
Unspecified	261 (1.0)

Abbreviation: ASD, autism spectrum disorder.

^a^ Indicates age at first diagnosis of ASD.

^b^ West includes Washington, Oregon, Idaho, Montana, Wyoming, Colorado, Utah, Nevada, and California; Midwest, Minnesota, Wisconsin, Michigan, Ohio, Indiana, Illinois, Iowa, Missouri, Kansas, Nebraska, South Dakota, and North Dakota; Southwest, Oklahoma, Texas, New Mexico, and Arizona; Southeast, West Virginia, Delaware, Maryland, Washington, DC, Virginia, North Carolina, South Carolina, Kentucky, Tennessee, Georgia, Florida, Alabama, Mississippi, Louisiana, and Arkansas; Northeast, New Jersey, Pennsylvania, New York, Connecticut, Rhode Island, Massachusetts, New Hampshire, Vermont, and Maine; and noncontiguous, Alaska, Hawaii, Puerto Rico, and Virgin Islands.

### Trends in Medication Use

**Figure 2.**
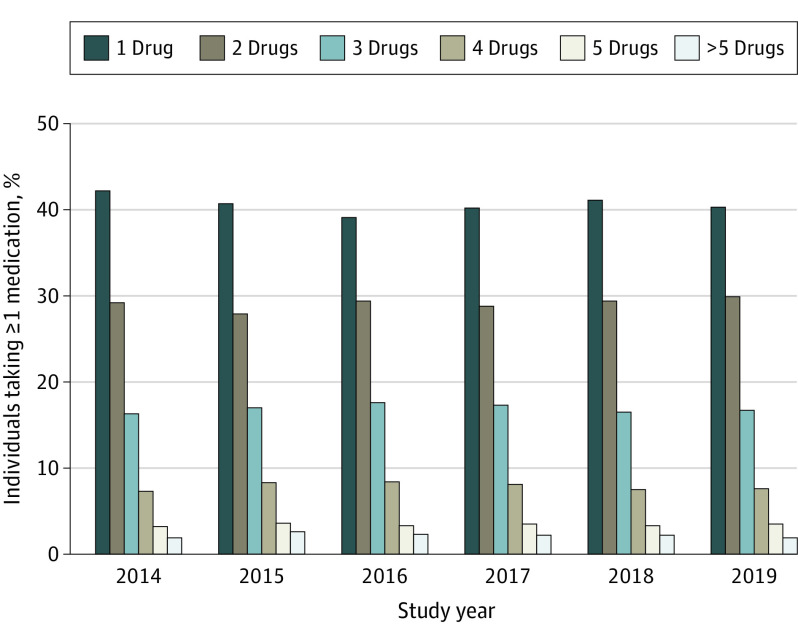
Frequency of Prescriptions by Year and Regimen Values are reflected over time. The number of drugs includes only the 24 under examination in this study.

In any given year, most individuals were prescribed only 1 of the drugs assessed in our study (40.6%), and a decreasing number were prescribed a drug regimen of 2 (29.1%), 3 (16.9%), 4 (7.9%), or 5 (3.4%) study medications (Figure 1 and Figure 2; the frequency of specific combination drug regimens is provided in the eFigure in the Supplement). Polypharmacy (≥3 medications at one time) ranged from 28.6% to 31.5%.

**Figure 3.**
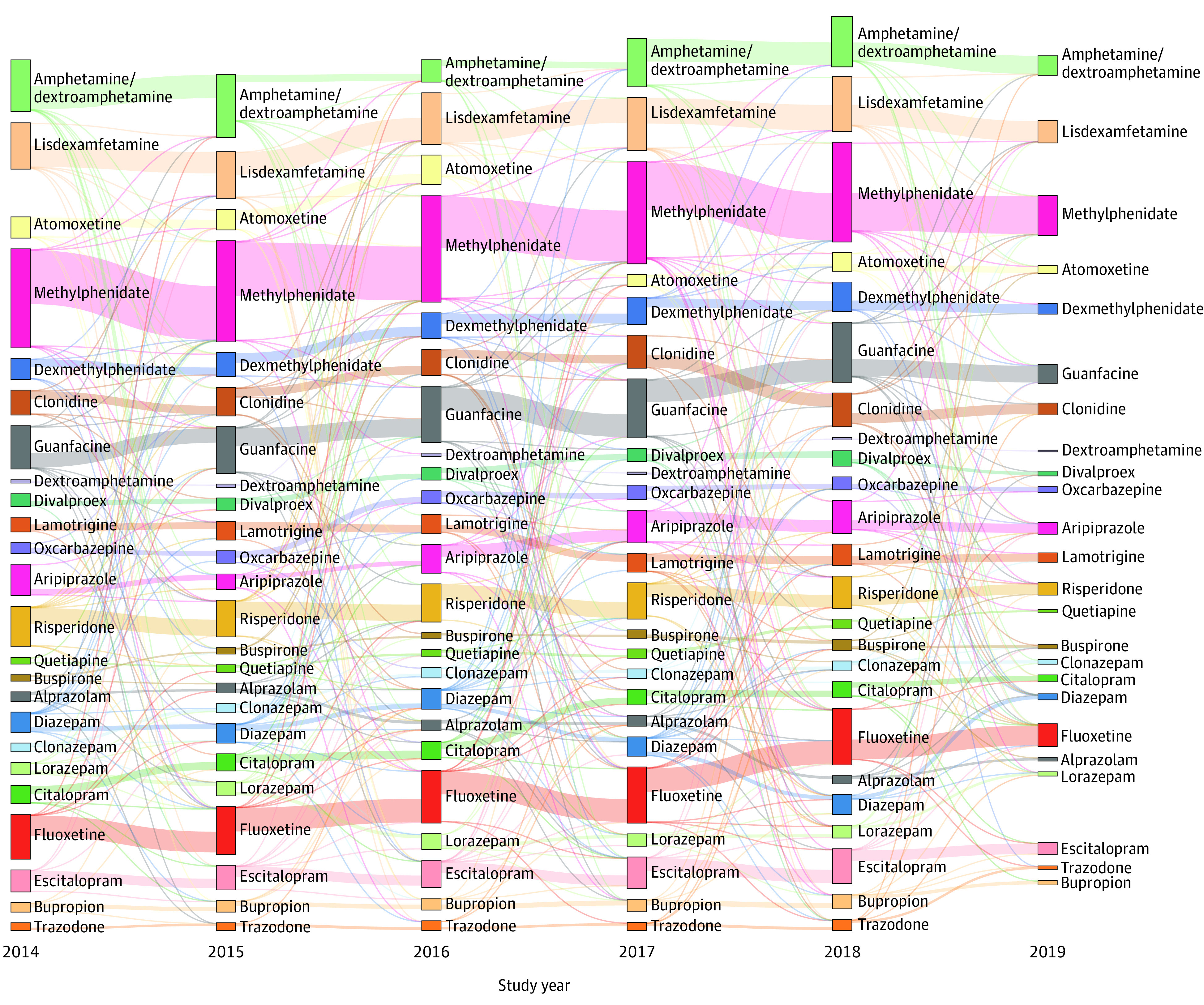
Trends in Prescription Drug Use Over Time This Sankey diagram^36^ only includes individuals who were prescribed a single drug in each of the listed years so the bars in each year are mutually exclusive. Medications are organized by drug class (eg, stimulants). The size of the vertical bars corresponds to the number of individuals exclusively prescribed that medication in that year. The lines between the vertical bars represent whether individuals took the same or another medication in the subsequent year. The width of the lines represents the relative proportion of individuals who continued taking a single drug in the following year (ie, if an individual used a drug not specified here or stopped taking a specified drug altogether, that individual would not be represented).

For individuals who were enrolled across the full 6-year study period, single-drug regimens showed frequent shifts annually (Figure 3). Individuals were prescribed medications within the same drug class (eg, fluoxetine hydrochloride to escitalopram oxalate) and then switched to other drug classes (eg, fluoxetine to aripiprazole) or had all medications discontinued. Despite variation in the specific individuals who used a certain medication at any one time, the total number of individuals prescribed a drug in our study during a given year stayed relatively consistent. For example, the total number of individuals prescribed methylphenidate shifted from 832 in 2014 to 850 in 2015, 899 in 2016, 863 in 2017, and 838 in 2018. Overall, the total number of individuals prescribed methylphenidate changed by only 0.7%.

### Comorbidity Analysis

**Figure 4.**
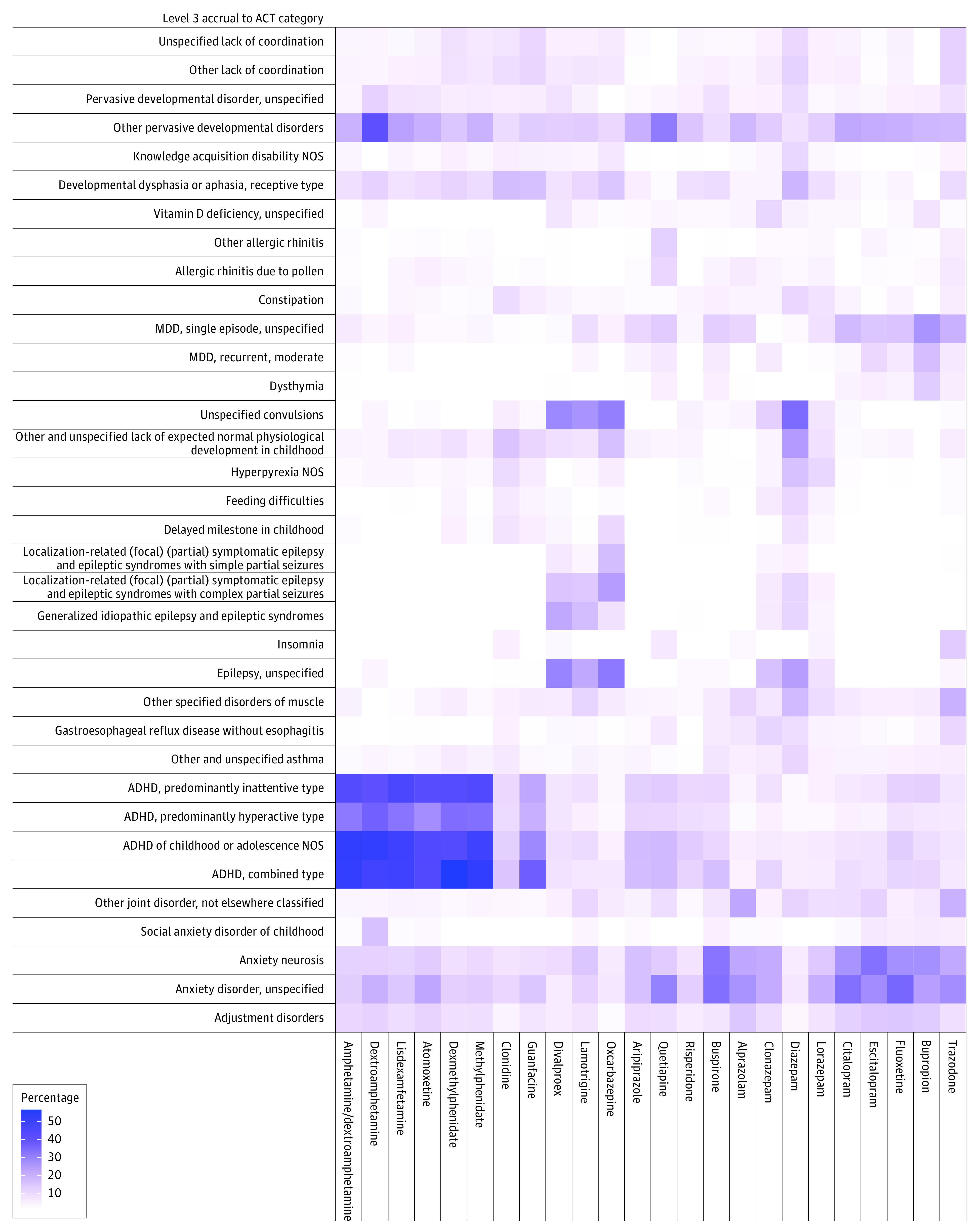
Heat Map of the Most Common Comorbidities for Each Drug of Interest Level 3 Accrual to Clinical Trials (ACT) categorizations are ordered by level 1 ACT category. The intensity of the color in each square (ie, the percentage) represents the relative proportion of individuals within each column (ie, a given prescription group) that also had the corresponding diagnostic categorization. Each column represents individuals in the study cohort taking only the corresponding medication during the 6-year study period. ADHD indicates attention-deficit/hyperactivity disorder; MDD, major depressive disorder; NOS, not otherwise specified.

Across all medication groups, the prevalence of co-occurring disorders ranged widely, depending on the type of disorder and the prescribed medication (Figure 4). In 2.1% of individuals given guanfacine, for example, there was an episode of major depressive disorder, whereas the same was true of 26.4% of individuals given bupropion hydrochloride. Similarly, the prevalence of anxiety disorder ranged from 6.2% (of those prescribed divalproex sodium) to 35.2% (of those given fluoxetine). The combined type of ADHD appeared in 4.9% of individuals with ASD treated with alprazolam to 56.4% given dexmethylphenidate hydrochloride. Finally, epilepsy was diagnosed in 1.1% of individuals administered escitalopram and 31.8% treated with oxcarbazepine.

Certain co-occurring conditions appeared to be associated with many of the medications examined in this study (Figure 4). In 15 of 24 medication groups assessed in this study, 15% or more of the individuals in the group had either an unspecified anxiety disorder, anxiety neurosis, or major depressive disorder (single episode). In 11 of 24 medication groups, 15% or more of the individuals had a form of ADHD (hyperactive, inattentive, and/or combined type).

Some medications were not as strongly associated with any particular co-occurring condition (Figure 4). In the following groups, there was a 10% or higher prevalence of many conditions: dextroamphetamine (11 co-occurring conditions), diazepam (16 co-occurring conditions), guanfacine (10 co-occurring conditions), lamotrigine (11 co-occurring conditions), quetiapine fumarate (10 co-occurring conditions), and trazodone hydrochloride (10 co-occurring conditions).

For patients taking antipsychotics, common comorbidities included combined type ADHD and anxiety disorder. Combined type ADHD was associated with 17.2% of those taking aripiprazole, 17.8% of those taking quetiapine, and 11.6% of those taking risperidone. Anxiety disorder was associated with 16.1% of those taking aripiprazole, 30.1% of those taking quetiapine, and 13.1% of those taking risperidone.

For patients taking stimulants, the prevalence of ADHD varied widely by medication and ADHD type. Combined type ADHD was associated with individuals taking the following ADHD medications: amphetamine (52.2%), atomoxetine hydrochloride (43.8%), dexmethylphenidate (56.4%), dextroamphetamine (48.0%), lisdexamfetamine dimesylate (49.1%), and methylphenidate (52.9%). Inattentive type ADHD was associated with a high proportion of patients taking amphetamine (43.3%), atomoxetine (42.3%), dexmethylphenidate (43.1%), dextroamphetamine (40.0%), lisdexamfetamine (45.6%), and methylphenidate (44.1%). In comparison, hyperactive type ADHD was associated with the following proportions of individuals taking ADHD medications: amphetamine (31.8%), atomoxetine (27.7%), dexmethylphenidate (34.0%), dextroamphetamine (36.0%), lisdexamfetamine (32.6%), and methylphenidate (33.7%).

## Discussion

To our knowledge, the present study is the first of its kind to examine both the comorbidities and use of medication in a longitudinal cohort of individuals with ASD in a population-based approach without preemptively constraining the analysis to a select few conditions or medication classes. This cohort study of medication prescriptions and comorbidities in 26 722 individuals with ASD during a 6-year period demonstrates significant variability in both the medication class prescribed and prescription frequency; only some of this variability can be explained by the management of specific comorbidities.

Previous analyses of the ASD population in the US have reported that individuals with ASD experience higher rates of psychiatric diagnoses and are more likely to be prescribed psychotropic medications compared with individuals without ASD.^2,37^ However, previous investigations have relied on a cross-sectional analysis of a single year or longitudinal analysis of a small cohort. Both are limited to a few select illnesses and have produced varying estimates for the prevalence of co-occurring conditions.^9,38^

Similarly, prior studies have often restricted analyses to predefined therapeutic medication classes. Even when restricted to psychotropic medications, estimated use rates have varied widely, from 27% to 79%.^26,37,39,40,41,42,43^ The wide range of estimates may be explained in part by our finding of frequent shifts in a given prescription for an individual from year to year, predominantly within rather than between medication classes (eg, a transition from fluoxetine to citalopram hydrobromide, rather than from a selective serotonin reuptake inhibitor to a stimulant). Possible drivers for shifting medications within a single therapeutic class may include patient preference, adverse effects, and cost considerations. In addition to changing medications, the decision to not renew a prescription and begin another may indicate differences in local prescribing patterns, changes in diagnostic trends, and ongoing challenges unique to the complex management of patients with ASD.

The wide variety of medications prescribed to individuals with ASD may be driven by clinical trends in the management of ASD and co-occurring symptoms and conditions. Recent recommendations from the American Academy of Pediatrics suggest that clinicians should investigate coexisting conditions in their patients with ASD to perhaps choose a behavioral rather than pharmacological intervention.^44^ However, recent work has found that relatively few children are receiving recommended behavioral therapies.^45^ Although there is no medical treatment for the core deficits of social communication and repetitive behavioral patterns in ASD, the American Academy of Pediatrics recommends that clinicians consider medications in the management of common comorbid conditions, including seizures, ADHD, anxiety disorders, mood disorders, and disruptive behavior disorders.^44^ The evidence base for interventions for children with ASD has been changing rapidly, and meta-analyses have found that high-intensity behavioral interventions may not be appropriate for all patients with ASD.^46^ Our findings suggest that clinicians may be increasingly using integrated approaches to treating patients with ASD and co-occurring conditions, and further work is necessary to determine the relative effects of pharmacotherapy vs behavioral interventions on outcomes in patients with ASD.

The present study suggests that clinicians are indeed incorporating pharmaceuticals into their management plans. Polypharmacy (defined as a regimen of ≥3 medications at a given time) was found to be common in this study population with ASD, ranging from 28.6% to 31.5% of individuals with ASD from 2014 to 2019. This estimate is higher than reports from prior longitudinal studies in solely pediatric populations with ASD. In children, reported estimates of psychotropic polypharmacy have ranged from 6.7% to 22%, with prescription rates even greater in individuals with comorbid conditions, such as ADHD.^37,47,48^ The higher rates of medication use in the present study may be secondary to the broader slate of common medications included in our analyses compared with prior studies. However, high rates of polypharmacy and medication transiency raise concerns about the efficacy of current medications in managing comorbidities as they occur in the context of ASD.

High rates of medication use may also reflect temporal trends in comorbidities; recent cross-sectional surveillance data from the Autism and Developmental Disabilities Monitoring Network found that the mean number of diagnosed comorbidities has also been rising.^49^ This trend has been explained by the increased awareness of ASD symptoms prompting earlier identification of ASD and more ASD diagnoses in the setting of milder clinical presentations.^49,50,51^ Further research is necessary to understand how long-term use of medications affects individuals with ASD and whether the frequent change in medication regimens is attributable to increasing diagnoses or preemptive concerns about adverse effects.

Notably, medication prescriptions in this study did not appear to be completely explained by associated comorbidities. The prevalence of co-occurring conditions varied widely depending on the medication used but did not always cluster as much as clinical guidelines might suggest.^44^ For example, 15% or more of individuals in 15 of 24 medication groups had a mood disorder, which is not the primary indication for several of these medications, including aripiprazole, atomoxetine, and quetiapine. Similarly, some medications (eg, diazepam, dextroamphetamine, and lamotrigine) appear to be weakly associated with many different comorbidities. These trends suggest that clinicians may be using these medications to treat ASD symptoms, even without diagnoses of comorbidities, or that pharmacologically treating these complex conditions is challenging and requires trials of several medications to achieve relief.

### Limitations

This study has some limitations. Although the study population includes individuals from across the US, the estimates of prescription frequency and comorbidity diagnoses may not be valid in populations outside of the US owing to possible differences in health insurance coverage, diagnostic criteria, and treatment guidelines.

Data validity can also be subject to misinformation and selection bias that arise from inconsistencies in reporting and availability of medical diagnoses and pharmacy claims data over time. Individuals with ASD who appeared to be taking no medications may, in fact, have been prescribed a less common medication that was not included in this study. Similarly, the prescription might not be recorded. Furthermore, individuals were identified as having ASD and other comorbidities using *ICD-9* and *ICD-10* codes without direct clinical assessment to validate the diagnoses.^52^ In addition, the limited number and use of validated diagnostic instruments to screen for comorbidities in individuals with ASD may contribute to underestimates in several diagnostic categories.^53^

In light of these limitations, we took careful steps to optimize study validity. We increased measure validity in the use of diagnostic codes as a proxy for true clinical diagnoses by restricting our cohort to individuals with ASD diagnostic codes recorded on at least 3 distinct occasions. Similarly, only the diagnostic codes recorded on at least 3 distinct occasions before a given medication prescription were included in the comorbidity analysis. In addition, we addressed concerns of coverage-related population instability (eg, job changes, health plan changes) in the comorbidity analysis by requiring continual enrollment throughout the study period.^54^ These strict inclusion criteria may have introduced minor selection bias that would have resulted in underestimates of the actual prevalence of ASD and the associated comorbidities.

## Conclusions

This cohort study demonstrates considerable variability and transiency in the use of prescription medications by US clinicians to manage symptoms and comorbidities associated with ASD. This study supports the importance of early and ongoing surveillance of patients with ASD and co-occurring conditions and offers clinicians insight on the targeted therapies most commonly used to manage co-occurring conditions. Future research and policy efforts are critical to assess the extent to which pharmacological management of comorbidities affects quality of life and functioning in patients with ASD, while continuing to optimize clinical guidelines, to ensure effective care for this growing population.

## Notes

Supplement. **eMethods 1.** Accrual to Clinical Trials (ACT) Ontology **eMethods 2.** The RECORD Statement: Checklist of Items, Extended From the STROBE Statement, That Should Be Reported in Observational Studies Using Routinely Collected Health Data **eTable.** Level 3 Diagnostic Codes Excluded From Comorbidity Analysis **eFigure.** Frequency of the Most Common Prescription Regimens of Individuals With ASD From 2014 to 2019
