# Supplementary material for: Medication Use in the Management of Comorbidities Among Individuals With Autism Spectrum Disorder From a Large Nationwide Insurance Database
Source: JAMA Pediatr. 2021 Jun 7;175(9):1–9. doi: 10.1001/jamapediatrics.2021.1329 (PMC8185632; doi:10.1001/jamapediatrics.2021.1329)
Supplement: Supplement. — eMethods 1. Accrual to Clinical Trials (ACT) Ontology eMethods 2. The RECORD Statement: Checklist of Items, Extended From the STROBE Statement, That Should Be Reported in Observational Studies Using Routinely Collected Health Data eTable. Level 3 Diagnostic Codes Excluded From Comorbidity Analysis eFigure. Frequency of the Most Common Prescription Regimens of Individuals With ASD From 2014 to 2019 [file jamapediatr-e211329-s001.pdf]

Supplementary Online Content

Feroe AG, Uppal N, Gutiérrez-Sacristán A, et al. Medication use in the management of comorbidities among individuals with autism spectrum disorder from a large nationwide insurance database. *JAMA Pediatr*. Published online June 7, 2021.
doi:10.1001/jamapediatrics.2021.1329

eMethods 1. Accrual to Clinical Trials (ACT) Ontology

eMethods 2. The RECORD Statement: Checklist of Items, Extended From the STROBE Statement, That Should Be Reported in Observational Studies Using Routinely Collected Health Data

eTable. Level 3 Diagnostic Codes Excluded From Comorbidity Analysis

eFigure. Frequency of the Most Common Prescription Regimens of Individuals With ASD From 2014 to 2019

This supplementary material has been provided by the authors to give readers additional information about their work.

© 2021 Feroe AG et al. *JAMA Pediatrics*.

eMethods 1. Accrual to Clinical Trials (ACT) Ontology

The ACT Ontology is a means of combining diagnostic codes from both the ICD-9 and ICD-10 systems and aggregating them into distinct levels for research purposes. A description of the current version of the ontology can be found here:

https://dbmi-pitt.github.io/ACT-Network/ontology.html.

There are nine levels of aggregation, in which Level 1 represents the broadest category and Level 9 represents the most granular (i.e. the specific ICD-9/ICD-10 code for a patient encounter). For example, an ICD-10 code for attention-deficit hyperactivity disorder (unspecified type) would have been aggregated as follows:

Example: ICD 10 code: F90.9 Attention-deficit hyperactivity disorder, unspecified type

Map to:

- ACT Level 1: Behavioral and emotional disorders with onset usually occurring in childhood and adolescence (f90-f98)
- ACT Level 2: Attention-deficit hyperactivity disorders
- ACT Level 3: Attention-deficit hyperactivity disorder of childhood or adolescence nos
- …

Present Study

In this study, the ACT Ontology was used to reorganize billing codes from a health insurer and capture broader diagnostic trends. Importantly, the ontology allowed for the comparison of diagnostic trends that spanned the transition from ICD-9 to ICD-10 coding systems. Level 3 of the ACT Ontology was chosen as the basis of comparison in this study in order to balance the tradeoff in detail from comparing the vast numbers of ICD and the broadest categorization offered by Level 1. For the study cohort, the number of distinct diagnostic categories varied by level as follows:

© 2021 Feroe AG et al. *JAMA Pediatrics*.

ICD10 Codes: 91,586

ACT Level 1: 226

ACT Level 2: 1,570

ACT Level 3: 7,686

ACT Level 4: 10,877

ICD9 Codes: 16,438

ACT Level 1: 230

ACT Level 2: 1,518

ACT Level 3: 7,199

ACT Level 4: 11,374

© 2021 Feroe AG et al. *JAMA Pediatrics*.

eMethods 2. The RECORD Statement: Checklist of Items, Extended From the STROBE Statement, That Should Be Reported in Observational Studies Using Routinely Collected Health Data

	Item No.	STROBE items	Location in manuscript where items are reported	RECORD items	Location in manuscript where items are reported
**Title and abstract**					
	1	(a) Indicate the study's design with a commonly used term in the title or the abstract (b) Provide in the abstract an informative and balanced summary of what was done and what was found	**(a) Lines 64-66**<br><br>**(b) Lines 54-97**	RECORD 1.1: The type of data used should be specified in the title or abstract. When possible, the name of the databases used should be included.<br><br>RECORD 1.2: If applicable, the geographic region and timeframe within which the study took place should be reported in the title or abstract.<br><br>RECORD 1.3: If linkage between databases was conducted for the study, this should be clearly stated in the title or abstract.	**1.1: Lines 1-2; 64-69**<br><br>**1.2: Lines 2; 60-69**<br><br>**1.3: N/A**
**Introduction**					
Background rationale	2	Explain the scientific background and rationale for the investigation being reported	**Lines 99-141**		
Objectives	3	State specific objectives, including any prespecified hypotheses	**Lines 138-147**		

© 2021 Feroe AG et al. *JAMA Pediatrics*.

Methods					
Study Design	4	Present key elements of study design early in the paper	**Lines 152-159**		
Setting	5	Describe the setting, locations, and relevant dates, including periods of recruitment, exposure, follow-up, and data collection	**Lines 161-166**		
Participants	6	*(a) Cohort study* - Give the eligibility criteria, and the sources and methods of selection of participants. Describe methods of follow-up<br>*Case-control study* - Give the eligibility criteria, and the sources and methods of case ascertainment and control selection. Give the rationale for the choice of cases and controls<br>*Cross-sectional study* - Give the eligibility criteria, and the sources and methods of selection of participants<br><br>*(b) Cohort study* - For matched studies, give matching criteria and number of exposed and unexposed<br>*Case-control study* - For matched studies, give matching criteria and the number of controls per case	**(a) Lines 151-166**<br><br>**(b) N/A**	RECORD 6.1: The methods of study population selection (such as codes or algorithms used to identify subjects) should be listed in detail. If this is not possible, an explanation should be provided.<br><br>RECORD 6.2: Any validation studies of the codes or algorithms used to select the population should be referenced. If validation was conducted for this study and not published elsewhere, detailed methods and results should be provided.<br><br>RECORD 6.3: If the study involved linkage of databases, consider use of a flow diagram or other graphical display to demonstrate the data linkage process, including the number of individuals with linked data at each stage.	**6.1: Lines 151-193, link to code in GitHub (lines 196-197)**<br><br>**6.2: Lines 162-166**<br><br>**6.3: N/A**
Variables	7	Clearly define all outcomes, exposures, predictors, potential confounders, and effect modifiers.	**Lines 168-193**	RECORD 7.1: A complete list of codes and algorithms used to classify exposures, outcomes, confounders, and effect	**7.1: Link to code in GitHub**

© 2021 Feroe AG et al. *JAMA Pediatrics*.

		Give diagnostic criteria, if applicable.		modifiers should be provided. If these cannot be reported, an explanation should be provided.	**(lines 196-197); lines 164-166; Supplement 1**
Data sources/ measurement	8	For each variable of interest, give sources of data and details of methods of assessment (measurement).<br>Describe comparability of assessment methods if there is more than one group	**Lines 168-193**		
Bias	9	Describe any efforts to address potential sources of bias	**Lines 161-166; 184-187, 189-193 (requirement of three unique diagnostic codes for a given condition; obtaining diagnostic data two years prior to pharmacy data for comorbidity analysis)**		
Study size	10	Explain how the study size was arrived at	**Lines 151-166, Figure 1 workflow diagram**		
Quantitative variables	11	Explain how quantitative variables were handled in the analyses. If applicable, describe which groupings were chosen, and why	**Lines 168-193**		

© 2021 Feroe AG et al. *JAMA Pediatrics*.

Statistical methods	12	(a) Describe all statistical methods, including those used to control for confounding<br>(b) Describe any methods used to examine subgroups and interactions<br>(c) Explain how missing data were addressed<br>(d) *Cohort study* - If applicable, explain how loss to follow-up was addressed<br>*Case-control study* - If applicable, explain how matching of cases and controls was addressed<br>*Cross-sectional study* - If applicable, describe analytical methods taking account of sampling strategy<br>(e) Describe any sensitivity analyses	**N/A**		
Data access and cleaning methods		..		RECORD 12.1: Authors should describe the extent to which the investigators had access to the database population used to create the study population.<br><br>RECORD 12.2: Authors should provide information on the data cleaning methods used in the study.	**12.1: Lines 375-376**<br><br>**12.2: Lines 152-193; Figure 1 workflow diagram**
Linkage		..		RECORD 12.3: State whether the study included person-level, institutional-level, or other data linkage across two or more databases. The methods of linkage and methods of linkage quality evaluation should be provided.	**12.3: Lines 151-166**

© 2021 Feroe AG et al. *JAMA Pediatrics*.

Results					
Participants	13	(a) Report the numbers of individuals at each stage of the study (*e.g.*, numbers potentially eligible, examined for eligibility, confirmed eligible, included in the study, completing follow-up, and analysed)<br>(b) Give reasons for non-participation at each stage.<br>(c) Consider use of a flow diagram	**(a) Lines 201-209**<br><br>**(b) Figure 1 workflow diagram**<br><br>**(c) Figure 1 workflow diagram**	RECORD 13.1: Describe in detail the selection of the persons included in the study (*i.e.,* study population selection) including filtering based on data quality, data availability and linkage. The selection of included persons can be described in the text and/or by means of the study flow diagram.	**13.1: Lines 201-209; Figure 1 workflow diagram**
Descriptive data	14	(a) Give characteristics of study participants (*e.g.*, demographic, clinical, social) and information on exposures and potential confounders<br>(b) Indicate the number of participants with missing data for each variable of interest<br>(c) *Cohort study* - summarise follow-up time (*e.g.*, average and total amount)	**(a) Lines 201-209; Table 1**<br><br>**(b) N/A**<br><br>**(c) N/A**		
Outcome data	15	*Cohort study* - Report numbers of outcome events or summary measures over time<br>*Case-control study* - Report numbers in each exposure category, or summary measures of exposure<br>*Cross-sectional study* - Report numbers of outcome events or summary measures	**Lines 211-261**		

© 2021 Feroe AG et al. *JAMA Pediatrics*.

Main results	16	(a) Give unadjusted estimates and, if applicable, confounder-adjusted estimates and their precision (e.g., 95% confidence interval). Make clear which confounders were adjusted for and why they were included<br>(b) Report category boundaries when continuous variables were categorized<br>(c) If relevant, consider translating estimates of relative risk into absolute risk for a meaningful time period	**N/A (descriptive statistics only)**		
Other analyses	17	Report other analyses done—e.g., analyses of subgroups and interactions, and sensitivity analyses	**Lines 228-261 (Figure 4, heatmap)**		
**Discussion**					
Key results	18	Summarise key results with reference to study objectives	**Lines 264-269**		
Limitations	19	Discuss limitations of the study, taking into account sources of potential bias or imprecision. Discuss both direction and magnitude of any potential bias	**Lines 333-356**	RECORD 19.1: Discuss the implications of using data that were not created or collected to answer the specific research question(s). Include discussion of misclassification bias, unmeasured confounding, missing data, and changing eligibility over time, as they pertain to the study being reported.	**19.1: Lines 334-346**

© 2021 Feroe AG et al. *JAMA Pediatrics*.

Interpretation	20	Give a cautious overall interpretation of results considering objectives, limitations, multiplicity of analyses, results from similar studies, and other relevant evidence	**Lines 271-330; 358-365**		
Generalisability	21	Discuss the generalisability (external validity) of the study results	**Lines 333-346**		
**Other Information**					
Funding	22	Give the source of funding and the role of the funders for the present study and, if applicable, for the original study on which the present article is based	**N/A, no source of funding to report related to the present study**		
Accessibility of protocol, raw data, and programming code		..		RECORD 22.1: Authors should provide information on how to access any supplemental information such as the study protocol, raw data, or programming code.	**22.1: Link to code in GitHub (lines 196-197); Supplemental Materials included**

*Reference: Benchimol EI, Smeeth L, Guttmann A, Harron K, Moher D, Petersen I, Sørensen HT, von Elm E, Langan SM, the RECORD Working Committee. The REporting of studies Conducted using Observational Routinely-collected health Data (RECORD) Statement. *PLoS Medicine* 2015; in press.

*Checklist is protected under Creative Commons Attribution (CC BY) license.

© 2021 Feroe AG et al. *JAMA Pediatrics*.

eTable. Level 3 Diagnostic Codes Excluded From Comorbidity Analysis
Autistic disorder
Encounter for newborn, infant and child health examinations
Motorized bicycle
Other unknown and unspecified cause of morbidity or mortality
Need for prophylactic vaccination and inoculation, Influenza
Bus occupant injured in transport accident (v70-v79)
Encounter for other specified aftercare
Other long term (current) drug therapy
Body mass index (bmi) pediatric
Pharyngitis (acute) nos
Acute upper respiratory infection, unspecified
Acne vulgaris
Hyperlipidemia, unspecified
Encounter for adult periodic examination (annual) (physical) and any associated laboratory and radiologic examinations
These codes were excluded from analysis since they either represented inclusion criteria (i.e. ‘Autistic disorder’), codes related to clinical encounters, or disease states not of specific clinical interest in the ASD population.

© 2021 Feroe AG et al. *JAMA Pediatrics*.

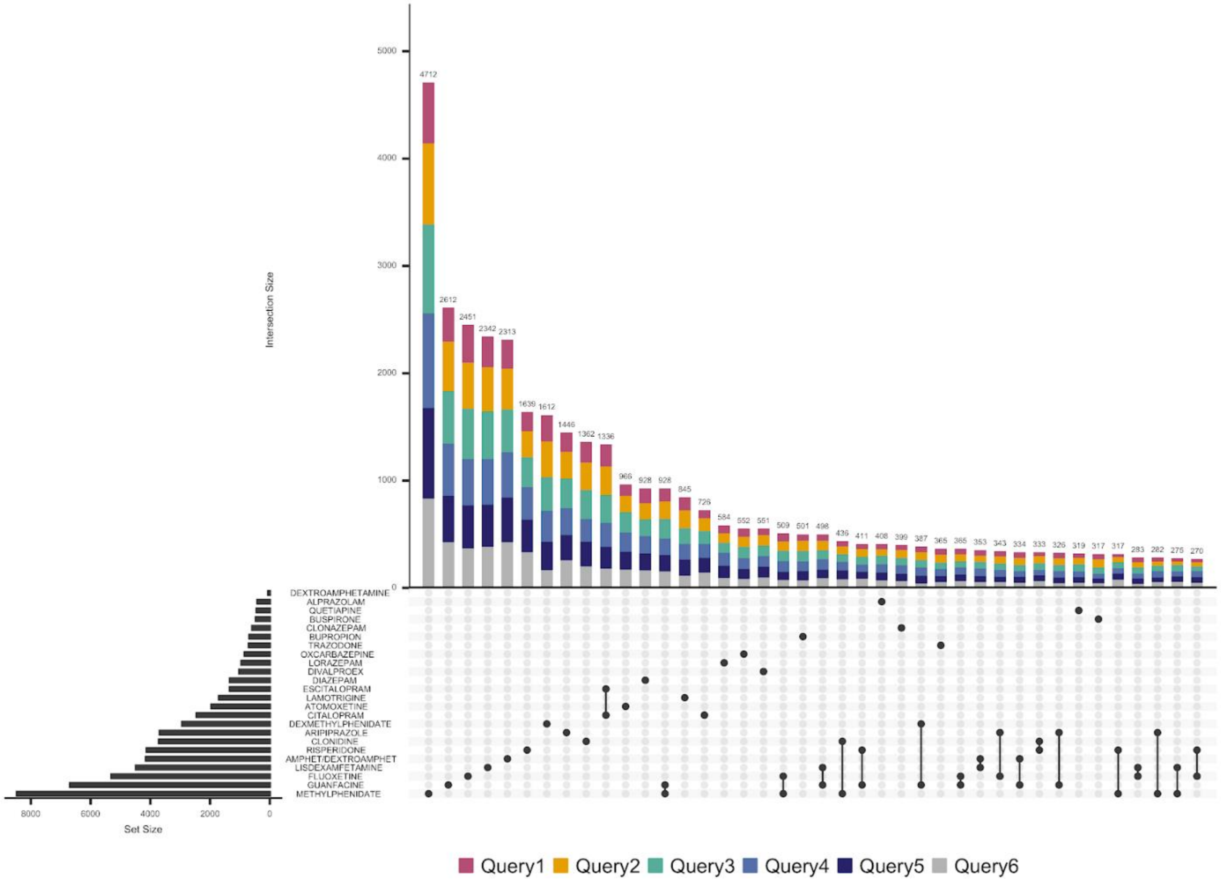

eFigure. Frequency of the Most Common Prescription Regimens of Individuals With ASD From 2014 to 2019

Each bar corresponds to a particular medication combination with a set size indicated in the intersection matrix. The colored segment of each bar represents the number of individuals taking the medication combination in the respective year. This visualization was created using UpSetR (InfoVis 2014).[55]

© 2021 Feroe AG et al. *JAMA Pediatrics*.
